# *Notes from the Field*: Follow-Up Assessment 1 Year After a Chemical Exposure Investigation — Winnebago County, Illinois, July–August 2022

**DOI:** 10.15585/mmwr.mm7203a6

**Published:** 2023-01-20

**Authors:** Ahlia Sekkarie, Peter DeJonge, Sandra Martell, DNP, Sarah Patrick, Motria Caudill, D. Kevin Horton, Maureen Orr, Stacey Konkle

**Affiliations:** ^1^Epidemic Intelligence Service, CDC; ^2^Winnebago County Health Department, Rockford, Illinois; ^3^Illinois Department of Public Health; ^4^Agency for Toxic Substance and Disease Registry.

On June 14, 2021, an industrial manufacturing facility in Winnebago County, Illinois caught fire and released smoke, dust, and debris, requiring evacuation of the area in the vicinity of the facility for 4 days. Following the emergency response, the Illinois Department of Public Health (IDPH) and Winnebago County Health Department (WCHD) requested assistance from the Agency for Toxic Substances and Disease Registry (ATSDR) to conduct a community Assessment of Chemical Exposure (ACE). That assessment found that almost one half of respondents reported symptoms during the 2 weeks after the fire (*1*).

One year after the fire, IDPH and WCHD invited ATSDR to conduct a follow-up ACE investigation to assess ongoing health impacts. WCHD and ATSDR emailed a modified survey to all 2,030 previous 2021 survey respondents, through the existing electronic system, to collect information related to ongoing exposure and mental and physical health symptoms. This investigation team also conducted a total of 22 semistructured interviews to collect open-ended responses to questions regarding mental health symptoms and community needs. Nine residents of a neighborhood adjacent to the fire site were interviewed in-person and 13 survey respondents who expressed interest in participating were interviewed by phone. 

Among the 2,030 previous survey respondents, 33% (676) completed the follow-up survey. In the follow-up survey, 39% (265) of respondents reported new or worsening mental health symptoms since the fire, among whom 98% were still experiencing these symptoms 1 year after the fire; 59% (400) reported new or worsening physical health symptoms, among whom 90% were still experiencing these symptoms 1 year after the fire.

Semistructured interviews enriched the quantitative information from the electronic survey and revealed themes related to anxiety, disappointment in communication, and overall poor mental health. More formal qualitative analysis is underway; however, preliminary findings suggest that residents were unable to easily access information related to environmental exposures or fire-site cleanup efforts. In addition, when information was available, respondents found it to be overly technical and difficult to interpret.

The semistructured interviews also provided an opportunity for residents to offer unprompted remarks. Residents reported avoidance of previous activities, such as gardening, because of concerns about fire-related contaminants. This information provides local authorities with specific actions such as targeted community environmental risk education.

This follow-up ACE investigation provided informative and immediately actionable data. Communities that have experienced a similar type of fire or environmental disaster would benefit from a consolidated source of information, summarized in easily understandable, plain language. The CDC Clear Communication Index is a set of research-based criteria that can be used to craft messages in an effective, interpretable way, especially for complex scientific information related to toxic substances (*2*). One limitation of this analysis is that data collected following disasters might be biased toward more reported adverse outcomes than those representative of the overall community (*3*).

Lessons learned from the follow-up survey are being applied to both the chemical fire and other environmental exposures such as per- and polyfluoroalkyl substances in water systems in Winnebago County. WCHD is working with IDPH and the Illinois Environmental Protection Agency on communications about chemical exposures that incorporate the concepts highlighted by this qualitative study including transparency about what is unknown and simple communications based on the CDC Clear Communication Index.

This investigation documented persistent mental and physical health symptoms reported among residents 1 year after a chemical fire, highlighted the importance of clear and accessible public health communications (*4*), and informed local authorities about previously unrecognized concerns. A follow-up ACE investigation for environmental disaster events by public health authorities can help gauge long-term health concerns and demonstrate ongoing investment in the wellbeing of affected communities.
